# Untargeted metabolomics to expand the chemical space of the marine diatom *Skeletonema marinoi*

**DOI:** 10.3389/fmicb.2023.1295994

**Published:** 2023-12-05

**Authors:** Mahnoor Zulfiqar, Daniel Stettin, Saskia Schmidt, Vera Nikitashina, Georg Pohnert, Christoph Steinbeck, Kristian Peters, Maria Sorokina

**Affiliations:** ^1^Faculty of Chemistry and Earth Sciences, Institute for Inorganic and Analytical Chemistry, Friedrich Schiller University Jena, Jena, Germany; ^2^Cluster of Excellence Balance of the Microverse, Friedrich Schiller University Jena, Jena, Germany; ^3^iDiv - German Centre for Integrative Biodiversity Research, Halle-Jena-Leipzig, Leipzig, Germany; ^4^Geobotany and Botanical Gardens, Martin-Luther University of Halle-Wittenberg, Halle, Germany; ^5^Institute of Plant Biochemistry, Leibniz Institute of Plant Biochemistry, Halle, Germany; ^6^Pharmaceuticals Division, Research & Development, Data Science and Artificial Intelligence, AG Bayer, Berlin, Germany

**Keywords:** diatom, *Skeletonema marinoi*, *Skeletonema*, untargeted metabolomics, metabolome annotation, chemical classification

## Abstract

Diatoms (Bacillariophyceae) are aquatic photosynthetic microalgae with an ecological role as primary producers in the aquatic food web. They account substantially for global carbon, nitrogen, and silicon cycling. Elucidating the chemical space of diatoms is crucial to understanding their physiology and ecology. To expand the known chemical space of a cosmopolitan marine diatom, *Skeletonema marinoi*, we performed High-Resolution Liquid Chromatography-Tandem Mass Spectrometry (LC-MS^2^) for untargeted metabolomics data acquisition. The spectral data from LC-MS^2^ was used as input for the Metabolome Annotation Workflow (MAW) to obtain putative annotations for all measured features. A suspect list of metabolites previously identified in the *Skeletonema* spp. was generated to verify the results. These known metabolites were then added to the putative candidate list from LC-MS^2^ data to represent an expanded catalog of 1970 metabolites estimated to be produced by *S. marinoi*. The most prevalent chemical superclasses, based on the ChemONT ontology in this expanded dataset, were organic acids and derivatives, organoheterocyclic compounds, lipids and lipid-like molecules, and organic oxygen compounds. The metabolic profile from this study can aid the bioprospecting of marine microalgae for medicine, biofuel production, agriculture, and environmental conservation. The proposed analysis can be applicable for assessing the chemical space of other microalgae, which can also provide molecular insights into the interaction between marine organisms and their role in the functioning of ecosystems.

## 1 Introduction

Unicellular microalgae, forming the phytoplankton, are primary producers in the marine food web, contributing to biodiversity and affecting the biogeochemical cycles, particularly the oceanic carbon cycling (Field et al., 1998; Litchman et al., 2015; Qian et al., 2016). Among the phytoplankton community, diatoms (Bacillariophyceae) are found in the euphotic layer of the ocean and have the largest contribution to the aquatic biosphere (Malviya et al., 2016). These eukaryotic microalgae survive under extreme environmental conditions such as varying light, temperature, nutrition, and salinity. They produce unique natural products (NP) with distinct biological activities displaying various chemical classes (Stonik and Stonik, 2015). As the primary producers, diatoms serve as a substantial biological source that can generate diverse natural products with applications in various NP-driven industries, such as food technology, cosmetics, biofuel, nanotechnology, and drug discovery (Lordan et al., 2011; Hildebrand et al., 2012; Martins et al., 2014; Khan et al., n.d.). However, the marine diatom community remains a large reservoir of unknown natural products (Nieri et al., 2023).

To expand the record of identified marine natural products or metabolites, we used *Skeletonema marinoi* (NCBI Taxonomy ID: 267567) as a model organism for diatoms. The strain used was taxonomically revised from *Skeletonema costatum* (Sarno et al., 2005). *S. marinoi* is a marine unicellular, centric diatom that lives in chain-like colonies consisting of up to 30 cells, with 1–2 chloroplasts in each cell located near the siliceous cell wall or frustule (Sarno et al., 2005). *Skeletonema marinoi* is considered a non-toxic species and generally safe (Silva et al., 2022). *S. marinoi* has been evolutionarily successful, and its genome has been able to encode certain bacterial, plant, and animal-like metabolic pathways (Di Dato et al., 2017). It can also adapt to varying environmental conditions (Milito et al., 2020). Given the optimal availability of nutrients, *S. marinoi* produces massive phytoplankton algal blooms in the coastal regions (Godhe and Härnström, 2010), releasing oxylipins that act as toxins for predators and other phytoplankton members (Fontana et al., 2007b; Ribalet et al., 2007). Although much effort has been made toward the chemical characterization of lipids in *S. marinoi*, as they impact growth, reproduction, stress response, and defense mechanism in the diatoms (Rousch et al., 2003; Fontana et al., 2007a; Popovich et al., 2012; Sayanova et al., 2017), much of its metabolome is still unexplored compared to other model species.

Mass spectrometry (MS)-based metabolomics is the key technology for detecting these secondary metabolites and covers the analysis of compounds with distinct structural heterogeneity (Bauermeister et al., 2021). In this study, we performed the metabolome annotation of the mass spectrometry data obtained from *S. marinoi* to comprehend the chemical composition of the primary and specialized metabolites produced by the diatom. We annotated molecular structures, chemical classes, and molecular formulae to MS features obtained with High-resolution Liquid Chromatography-Electrospray Ionization-Tandem Mass Spectrometry (LC-ESI-Orbitrap MS^2^) using Metabolome Annotation Workflow (MAW) (Zulfiqar et al., 2023). The findings of this study contribute to the expansion of known marine natural products derived from *S. marinoi*.

## 2 Methods and materials

### 2.1 Suspect list development and curation

For this study (Zulfiqar, 2022, 2023a,b), a suspect list was generated manually from different databases and literary resources (not considering the extraction and acquisition methods) to represent a collection of known metabolites produced by *Skeletonema marinoi* and its phylogenetically closely related species, *Skeletonema costatum*. The list contained primary metabolites, as well as the secondary metabolites that have been reported in *S. marinoi* and *S. costatum*. The 893 compounds in the list were collected and assembled from databases such as KEGG (Kanehisa and Goto, 2000; Kanehisa, 2019; Kanehisa et al., 2023), ChEBI (Hastings et al., 2016), PubChem (Kim et al., 2021), MetaCyc (Caspi et al., 2014), UniProtKB (The UniProt Consortium, 2021), BRENDA (Chang et al., 2021), LOTUS (Rutz et al., 2022), and several publications (Derenbach and Pesando, 1986; Pennington et al., 1988; Brown and Jeffrey, 1995; Anning et al., 2000; Ianora et al., 2004; Fontana et al., 2007a; Ribalet et al., 2007; Snoeijs et al., 2011; Vidoudez and Pohnert, 2012; Lauritano et al., 2015; Orefice et al., 2016; Di Dato et al., 2017; Amato et al., 2018; Jing et al., 2019; Smerilli et al., 2019; Cardoso et al., 2020; Gallo et al., 2020; Russo et al., 2021; Nikitashina et al., 2022). For curation and additional metadata on each entry, PubChemPy (Swain, n.d.) and RDKit (Landrum, 2016) were used by adding any missing synonyms, IUPAC names, and molecular weights. Entries with no identifier or structural notation were discarded. Lastly, pybatchclassyfire (Allard, 2020) was used to add classification based on ChemONT 2.1 (subclass, class, superclass) to the suspect list using SMILES (Djoumbou Feunang et al., 2016).

### 2.2 Sample preparation and metabolite extraction

For the whole metabolome extraction from LC-MS samples, methanol, ethanol, chloroform, acetonitrile, and water were used as reagents. *S. marinoi* RCC 75 strain used in this study was obtained from Roscoff Culture Collection (Roscoff, France). These cultures were grown in 40 mL artificial seawater (ASW) inside 50 mL cell culture flasks. The cultures were kept at the optimum conditions for the growth of this strain under a standard temperature of 13°C with 55–65 μmol photons m^−2^ s^−1^ lighting, keeping the 14:10 h light:dark regime. The cultures were incubated in a shaking incubator at 80 rpm. Cultures were collected on Whatman GF/C filters with 1.2 μm pore size (GE Healthcare, US) under vacuum (750 mbar). These conditions provide a filtering speed of ~1.5 mL/min. The filters were then submerged in an ice-cold extraction mixture of methanol:ethanol:chloroform, 1:3:1, v:v:v. Samples were ultrasonicated for 10 min, and the solutions were transferred to new Eppendorf tubes without the filters. The Eppendorf tubes with the solutions were then centrifuged at 30,000 g at 4°C for 15 min. The aliquots for LC-MS analyses were separated and evaporated using a vacuum. Samples with aliquots that contained 3 × 10E6 cells were prepared. Media blanks included the average volume for the culture's samples. The volumes varied from 25 to 40 μL depending on cell number in the cultures. The dried samples were stored in argon at −24°C. For LC-MS analysis, the dried samples were dissolved in 150 μL of the mixture of methanol:acetonitrile:water, 5:9:1, v:v:v.

### 2.3 LC-MS^2^ measurement

Samples separation with chromatography was performed on the SeQuant ZIC-HILIC column (5 μm, 200 Å, 150 × 2.1 mm, Merck, Germany) with a flow rate of 0.6 mL/min. The samples were measured with the Dionex Ultimate 3000 system coupled to a Q-Exactive Plus Orbitrap mass spectrometer (Thermo Scientific, Bremen, Germany). Using electrospray ionization, the molecular ions were obtained in both positive and negative modes, with a scan range of 80–1,200 m/z. The duration of the method was 12.5 min, and MS runtime ranged from 0.5–9 min. The separation was performed on the SeQuant ZIC-HILIC column (5 μm, 200 Å, 150 × 2.1 mm, Merck, Germany), equipped with SeQuant ZIC-HILIC Guard (20 × 2.1 mm, Merck, Germany). Solution A consisted of water with 2% acetonitrile and 0.1% formic acid, and solution B consisted of 90% acetonitrile with 10% water and 1 mmol/L-1 ammonium acetate. The flow rate was set to 0.6 mL/min-1. The sample was run through a solvent mixture comprising of 15% solvent A for a duration of 4 min. Afterward, the solvent was gradually increased to 100% solvent A at 5 min and was maintained at this level until 7 min. Following this, solvent B was gradually increased to 100% until 8 min and held at this level until 10 minutes. Finally, the solvent was gradually decreased to 15% solvent A at 10.5 min and maintained at this level until 12.5 min.

MS^1^ measurements were conducted with the following settings: automatic gain control target−3E6, the maximum ion injection time−200 ms, and scan range from 80 to 1,200 *m/z*, resolution 70,000. MS^2^ had the following settings: automatic gain control target 2E5, maximum ion injection time 100 ms, three-stepped normalized collision energy 15, 30, 45, and scan range from 80 to 1,200 *m/z*, resolution 70,000. MS^1^ and MS^2^ measurements were performed in parallel. A target list of all detected compounds was created with Compound Discoverer (Thermo Fisher Scientific) and was manually separated into 20 inclusion lists. We used retention time-based PRM and defined a narrow retention time window of 0.2 min for each feature in the inclusion list. All MS^2^ spectra were acquired within 20 consecutive injections of the same sample.

### 2.4 Metabolic profiling with Metabolome Annotation Workflow

**Figure 1:**
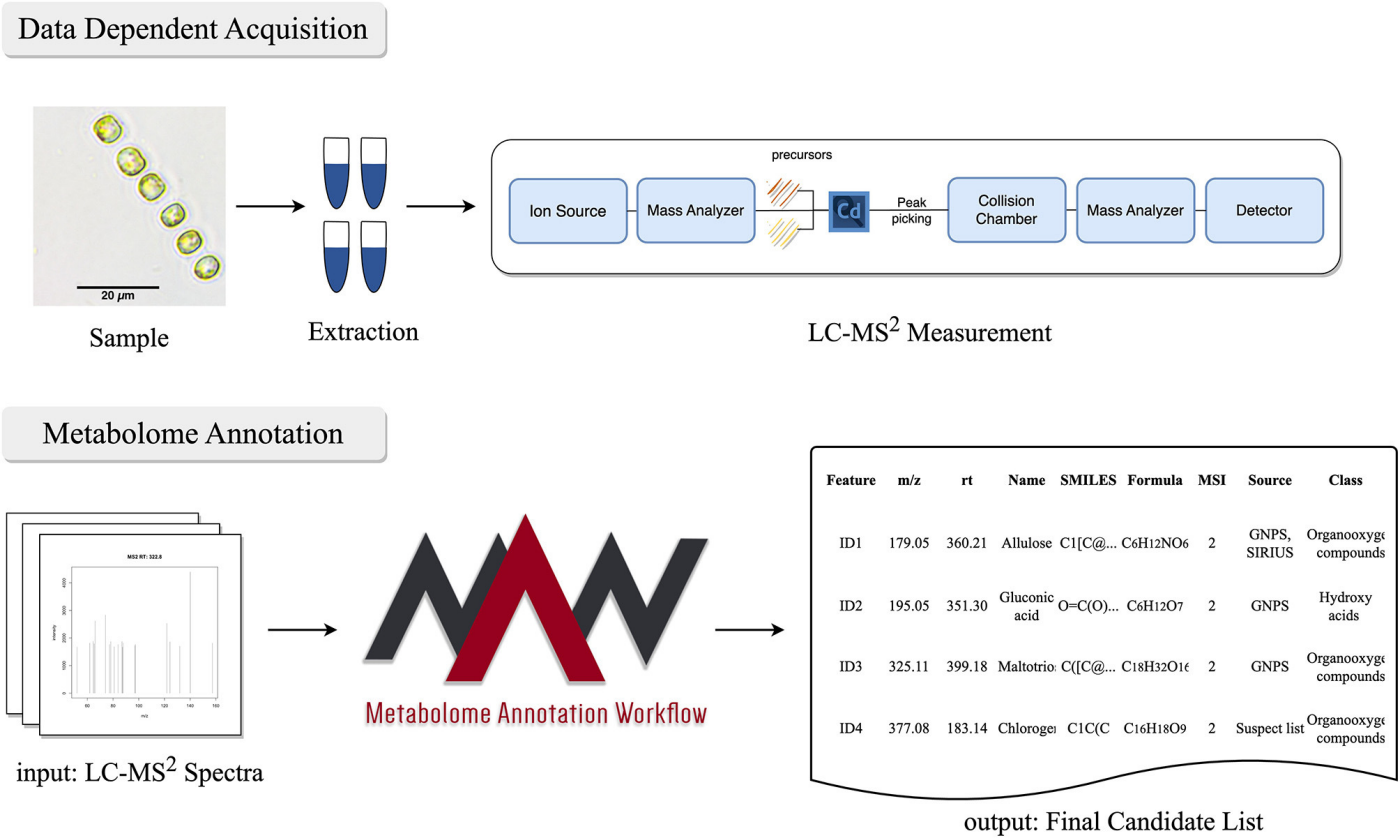
Workflow for untargeted mass spectrometry approach to acquire the metabolome from *Skeletonema marinoi*. After the sample collection and metabolic extraction, LC-MS^2^ measurements are performed using an orbitrap mass spectrometer. The data obtained from the LC-MS^2^ is then subjected to MAW, which performs the annotation of metabolites and generates a final list of metabolites extracted from the samples.

The RAW MS files obtained from the Orbitrap Mass Spectrometer were preprocessed using Compound Discoverer. The standard workflow (Untargeted Metabolomics with Statistics Detect Unknowns with ID Using Online Databases and mzLogic) was used with default options (Zulfiqar and Schmidt, 2021; Zulfiqar and Stettin, 2023a,b). The RAW MS^2^ files were converted to .mzML using Proteowizard MS-Convert Suite (Chambers et al., 2012). MAW (version 1.1) was used for dereplication against spectral and compound databases. For the spectral database dereplication, the input query spectra were matched against the experimental spectra from GNPS (Wang et al., 2016), Massbank (Horai et al., 2010), and HMDB (Wishart et al., 2007, 2018). For the compound database dereplication, we used SIRIUS4 (version 4.9.12) (Dührkop et al., 2015, 2019). SIRIUS performed the dereplication against the database “ALL,” which refers to all databases integrated within the software. CANOPUS (Dührkop et al., 2021) and ClassyFire (Djoumbou Feunang et al., 2016) predicted the chemical class of the candidates. The candidate selection module was used to list the most probable (high-scoring) candidates from spectral databases and SIRIUS. The sunburst function generated sunburst diagrams based on the chemical classes of top-ranking candidates. Figure 1 explains the untargeted approach to extracting the whole metabolome of the diatom and the application of MAW on the annotation of metabolites from the whole metabolome.

## 3 Results

### 3.1 Suspect list of known *S. marinoi* metabolites

A suspect list of known metabolites produced by *S. marinoi* and the closely related *S. costatum* was created using different databases and literature articles, described in the methodology section, for the purpose of dereplication of known metabolites annotated from the MS^2^ data acquired from the precursor masses (*m/z*) in the inclusion lists. The curated suspect list contained names, formulae, species, SMILES, InChIs, monoisotopic masses, PubChem identifiers, source databases and references, IUPAC names, synonyms, subclasses, classes, and superclasses of 893 compounds. For the classification of these metabolites, ClassyFire (Djoumbou Feunang et al., 2016) was used, which annotated 851 compounds from the suspect list. ClassyFire uses ChemONT, which is a structure-based taxonomy system used to annotate chemical compounds to a hierarchical system of classification. Metabolites belong to the class of organic compounds and can be categorized into more detailed superclasses (generic), classes (specific), and subclasses (highly specific). The most abundant superclasses in the suspect list, according to ChemONT, were organoheterocyclic compounds, organic acids and derivatives, organic oxygen compounds, and lipids and lipid-like molecules (see Supplementary Figure 1).

To comprehend the structural similarity among the known metabolites from the suspect list, we clustered together the chemically similar compounds from the abundant superclasses into a network (shown in Supplementary Figure 2). This chemical similarity network visually represents the structural similarity between different compounds, which can reveal joint functional regulation. A group of compounds in a cluster can represent a biochemical pathway or otherwise connected regulations. The chemical similarity network was created using an “all vs. all” approach, where the SMILES of all chemical structures in the suspect list were converted to Morgan Fingerprints (Morgan, 1965; Rogers and Hahn, 2010) and then were compared against one another using a Tanimoto similarity score, with a given threshold of ≥0.85. This calculation was carried out with RDKit (Landrum, 2016) using the MAW-defined function ChemMN (https://github.com/zmahnoor14/MAW/blob/main/cwl/Workflow_Python_Script_all.py#L2948). The chemical superclass with the highest internal similarity in the suspect list was the lipids and lipid-like molecules, exhibiting the polyunsaturated fatty acids (PUFA), polyunsaturated aldehydes (PUA), and precursor molecules such as acetyl CoA and malonyl CoA which drive the citric acid cycle and fatty acid biosynthesis (Kelly and Hughes, 2001). Organoheterocyclic compounds were also highly interconnected, representing different tetrapyrroles, such as chlorophyll A. The other two superclasses were amino acid derivatives and coenzymes and organic oxygen compounds belonging to polyphenols.

### 3.2 Structure annotation and classification of tandem mass spectrometry data from *S. marinoi*

To broaden the known chemical space of *S. marinoi*, we acquired untargeted metabolomics data from *S. marinoi* samples using High-Resolution Liquid Chromatography-Tandem Mass Spectrometry (HR-LC-MS^2^). The data was measured in both positive and negative modes, which resulted in 1,014 and 839 features, respectively, making a total of 1,853 distinct metabolic features with unique precursor masses (*m/z*) and retention time (seconds), out of which 1,153 were identified as unique metabolites. This difference in unique features and unique compounds can be attributed to the presence of various isotopes, adducts, and fragments from the same compound (Lu et al., 2020). The Metabolome Annotation Workflow was used to perform dereplication using spectral databases [GNPS (Wang et al., 2016), Massbank (Horai et al., 2010), and HMDB (Wishart et al., 2007, 2018)], and compound databases integrated with SIRIUS4 (Dührkop et al., 2015, 2019). The results were post-processed, and each distinct feature was assigned a list of putative structures with Metabolomics Standards Initiative (MSI) confidence levels of identification (Sumner et al., 2007; Schymanski et al., 2014; Zulfiqar et al., 2023). The standards used for this dataset included betaine, pipecolinic acid, cysteinolic acid, methionine sulfoxide, N,N-dimethylarginine, O-acetyl-L-carnitine, O-propanoyl-L-carnitine, O-butanoyl-L-carnitine, and isovalerylcarnitine, were annotated using MAW and were assigned MSI level 1 in the workflow article (Zulfiqar et al., 2023). Table 1 shows the number of unique features annotated within each MSI level.

**Table 1:** Number of features from the LC-MS^2^ data of *S. marinoi*, annotated with MAW, divided into different MSI levels.

**MSI level**	**Description**	**No. of features**
1	Structure identification with a standard	No standards were used here; the results for standards with MAW can be found in Zulfiqar et al. (2023)
2	Experimental spectral matching with one or more spectral databases	147
3	Tentative candidate structure	1,130
4	Molecular formula or chemical class	4
5	Only the spectral features from LC-MS^2^	572

To assess the metabolic profile of *S. marinoi* from LC-MS^2^ data, the high-scoring candidates with MSI levels 2 and 3 were analyzed. The annotations contained both primary metabolites, such as amino acids, and their derivatives (pipecolic acid, ornithine, carnitine), nucleic acids and analogs (adenosine, guanosine, cyclic AMP), carbohydrates (alpha-lactose and glucose-1-phosphate), fatty acids (eicosapentaenoic acid, myristic acid, stearic acid), and different other organic acids (gluconic acid, chlorogenic acid, and ascorbic acid). The composition of compound superclasses, classes, and subclasses in this experimental dataset is represented by a sunburst plot in Supplementary Figure 3. A total of 1,036 annotations were assigned a chemical superclass using CANOPUS (Dührkop et al., 2021) and ClassyFire. This dataset shows the same abundant superclasses as the suspect list.

### 3.3 Expansion of known metabolome of *S. marinoi*

**Figure 2:**
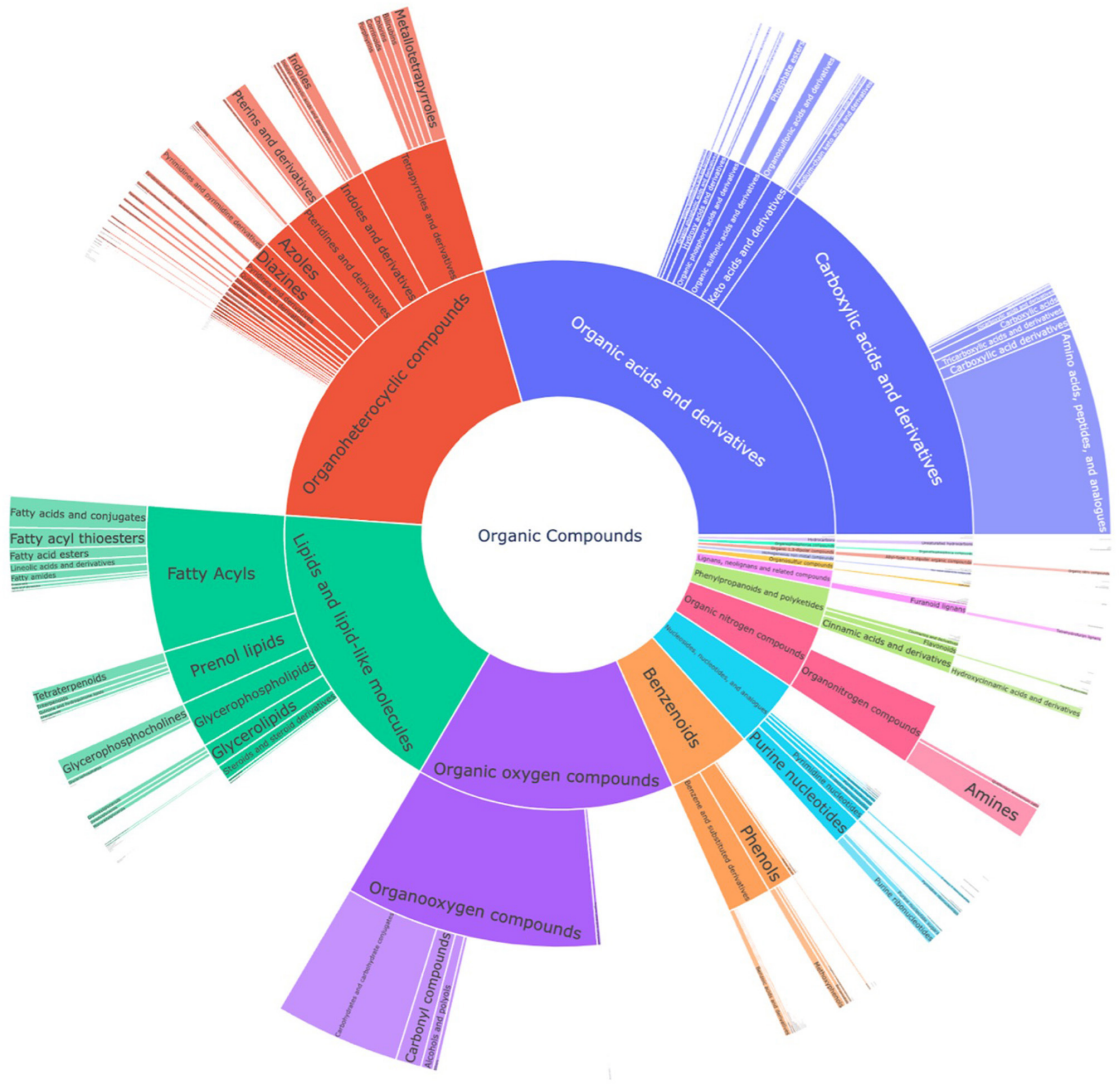
Sunburst plot for chemical classification of the union set from the suspect list and the LC-MS^2^ data from *S. marinoi*, which shows the expanded known metabolome of the diatom. The innermost section of the kingdom of organic compounds is classified into superclasses, classes, and subclasses. The most prevalent superclasses are organic acids and derivatives (28.1%), organoheterocyclic compounds (20.3%), lipids and lipid-like molecules (17.5%), and organic oxygen compounds (14.5%).

Lastly, the putative candidate list from the LC-MS^2^ data was compared with the suspect list to extract metabolites that were common in both datasets with a Tanimoto similarity score of 1, which led to 57 common compounds. The most prevalent class was carboxylic acids and derivatives, particularly amino acids and analogs, as most of the common metabolites were primary metabolites (Supplementary Figure 4). The compounds from the suspect list and LC-MS^2^ data were then combined into a single union set of metabolites, which resulted in 1997 compounds. Manual curation was performed to remove expectedly misannotated metabolites originating from plant, fungal, or animal origins, which then reduced the list to 1970 compounds that represented the expanded chemical space of compounds produced by *S. marinoi*. Figure 2 represents a sunburst plot of the chemical classification of 1,691 compounds from this expanded chemical space of compounds from *S. marinoi*.

**Figure 3:**
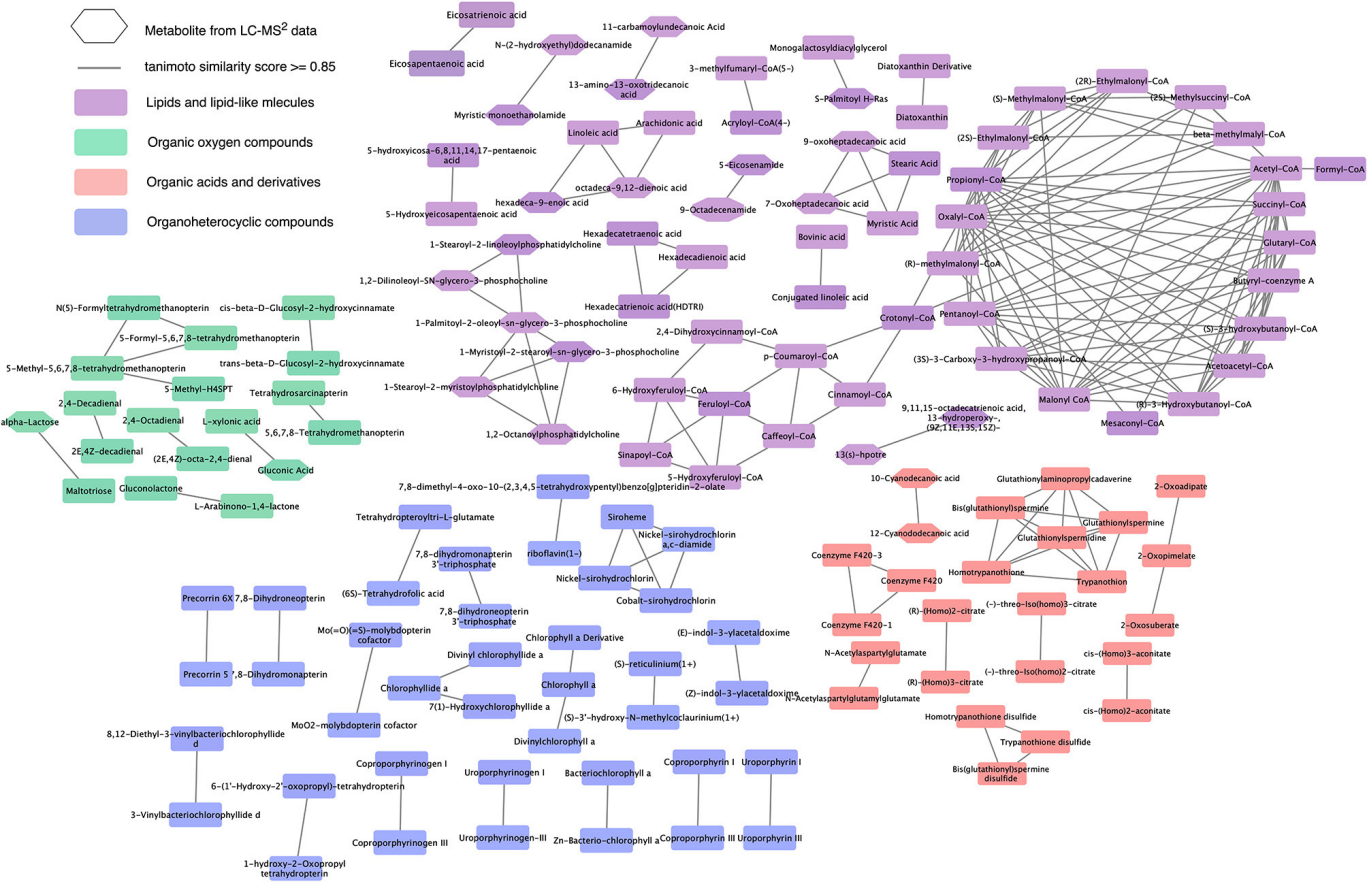
Expansion of chemical similarity network of the suspect list with the candidate structures from LC-MS^2^ data. This network represents the expanded chemical similarity space of the suspect list from Supplementary Figure 2; the new metabolites from the LC-MS^2^ data are represented with a hexagonal shape. This network shows the most abundant superclasses and the structural similarities between the metabolites from these superclasses.

The metabolites from the LC-MS^2^ data were incorporated into the chemical similarity network of compounds from the suspect list, shown in Supplementary Figure 2, to demonstrate the expansion in the chemical similarity, which indicated the metabolites previously not reported or missing from the chemical similarity network or the known metabolic pathways of the diatom (as depicted in Figure 3). The hexagonal-shaped metabolite nodes in Figure 3 represent the putative candidates from the LC-MS^2^ data. Figure 3 shows that the lipids and lipid-like molecules superclass had again the largest expansion representing newly added metabolites such as saturated oxo-fatty acid (SOFAs), fatty acid amides (FAAs), and glycerophospholipids. The organic oxygen compounds superclass expanded with building blocks of polysaccharides for the diatom cell wall. Cyanodecanoic and cyanododecanoic acids were added to the chemical similarity network of the organic acids and derivatives superclass.

## 4 Discussion

Marine natural product reservoirs are still partly underexplored/ unexploited (Nieri et al., 2023). The marine diatom *S. marinoi*, for example, has been extensively studied for its lipid metabolism; however, the chemical diversity has not yet been fully explored for other natural products. To understand the biochemical mechanisms occurring within ubiquitous diatoms, we expanded the chemical space of known metabolites from *S. marinoi* using a whole untargeted metabolomics approach. With our automated MAW, we annotated 69% of the metabolic features from the LC-MS^2^ data with a chemical structure or a molecular formula (see Table 1). We verified the presence of previously identified metabolites using the suspect list of compounds from *S. marinoi* as the source of known metabolites (see Supplementary Figures 1, 2). Here, we discuss metabolites annotated in this study, of which some have not been previously identified in *S. marinoi*.

### 4.1 Secondary metabolites in the expanded chemical space of *S. marinoi*

Using the LC-MS^2^ data, MAW annotated some of the known metabolites produced by *S. marinoi*, many of which were amino acids, analogs, and their derivatives such as betaine, pipecolic acid, 4-hydroxyproline, methionine sulfoxide, and *N*,*N*-dimethylarginine. Such polar compounds alleviate salt stress in *S. marinoi* and might act as osmoprotectants (Nikitashina et al., 2022). *S. marinoi* is also known to produce a rich portfolio of lipids and lipid-like molecules. With MAW, we annotated several polyunsaturated fatty acids (PUFA), such as eicosapentaenoic and eicosatrienoic acids, saturated fatty acids (SFA), such as myristic acid and stearic acid, and fatty acid esters (FAE) such as isovalerylcarnitine, propionylcarnitine, and butyrylcarnitine, which also act as stress protectants (Nikitashina et al., 2022). Being a photosynthetic organism, *S. marinoi* is also known to produce terpenoid pigments, such as astaxanthin and tocopherol, which were also verified with MAW (Silva et al., 2022).

MAW annotated 1,072 unique structures to metabolites that were not present in the suspect list and hence represented the expansion of known metabolites produced by *S. marinoi*. Only a few of these compounds show chemical similarity with the previously known natural products produced by *S. marinoi* (shown in Figure 3 as hexagonal nodes), among which most of the metabolites belonged to the lipids and lipid-like molecules superclass. Lipids in diatoms have been studied extensively due to their role as membrane constituents, regulators of gene expression, and sources of energy for the cells via fatty acid beta-oxidation (Yi et al., 2017; Vuong et al., 2020). In Figure 3, one of the chemical similarity clusters showed two saturated fatty acids (SFA)—myristic (14:0) and stearic acids (18:0)—and two saturated oxo-fatty acids (SOFA)−7-oxoheptadecanoic acid and 9-oxoheptadecanoic acid (derivatives of heptadecanoic acid). SOFAs are SFAs with a ketone group. This remains an understudied class of lipids with unknown pathways (Batsika et al., 2021) and hence cannot be linked back to the known diatom metabolism. However, the SFA heptadecanoic acid is present in algae, plants, and bacteria (Wikidata Contributors, n.d.). In a study done on *S. costatum*, the fatty acid profile in the stationary growth phase showed minute levels of heptadecanoic acid (Popovich et al., 2012), indicating the measurable presence of this SFA after the end of the cell growth period. Heptadecanoic acid has been exploited for its medicinal use against type 2 diabetes and skin cancer (Forouhi et al., 2014; Kathiresan, 2020). 4-Oxo sebacic acid, which is likely derived from sebacic acid (a dicarboxylic SFA), was also annotated using MAW; however, no previous records of sebacic acid in diatoms have been reported so far.

Another class of detected lipids is the fatty acid amide (FAA). A chemical similarity pair was formed between two FAA derivatives of myristic and lauric acid—myristic monoethanolamide and *N*-(2-hydroxyethyl)dodecanamide. Both are *N*-functionalized long-chain-acyl ethanolamines resulting from the condensation of the carboxy group of an SFA with the amino group of ethanolamine. *N*-(long-chain-acyl)ethanolamines are converted to SFAs by FAA hydrolases (Rhea-Fatty Acid Amide hydrolase ID: 45456, n.d.). Another pair of FAAs with high structural similarity was 5-eicosenamide (an unsaturated eicosanoic acid derivative) and 9-octadecanamide (a stearic acid derivative). Two other high-scoring examples (from Table 2) are hexadecanamide (derived from palmitic acid) and tetradecanamide (derived from myristic acid). Carboxamides also undergo conversion back to SFA. SFAs are present in the lipid profile of many diatoms (Pratiwi et al., 2009); however, there is no systematic research done on their FAA derivatives reported in this study. FAAs, such as oleamide from oleic acid, have anti-biofouling effects (Fiorini et al., 2020) on the accumulated microalgal biofilms on various surfaces, but this result has to be verified in the light of our finding that microalgae contribute themselves to the compound class.

**Table 2:** List of top-ranking metabolites (based on MAW candidate selection criterion) from the LC-MS^2^ data annotated with MAW from MSI levels 2 and 3.

**Precursor mass (m/z)**	**Retention time (s)**	**Molecular formula**	**Molecular structure**	**Chemical class**	**Name of the compound**
256.2635	46	C_16_H_33_NO	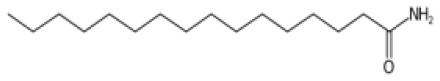	Fatty acyls	Hexadecanamide
162.0758	103	C_6_H_11_NO_4_	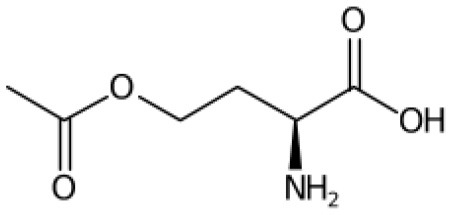	Carboxylic acids and derivatives	O-acetyl-L-homoserine
195.0510	351	C_6_H_12_O_7_	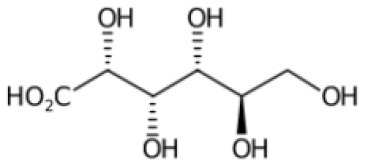	Hydroxy acids and derivatives	Gluconic acid
217.1046	57	C_10_H_16_O_5_	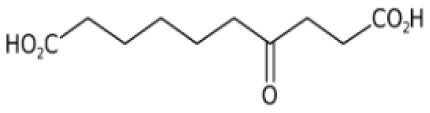	Carboxylic acids and derivatives	4-Oxosebacic acid
282.2790	46	C_18_H_35_NO	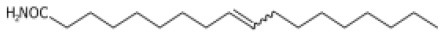	Fatty acyls	9-Octadecenamide
103.0503	390	C_3_H_6_N_2_O_2_	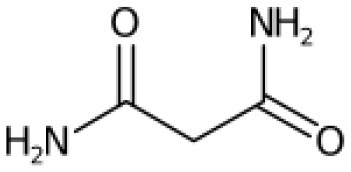	Organooxygen compound	Malonamide
228.2322	43	C_14_H_29_NO	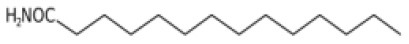	Fatty acyls	Tetradecanamide
149.0235	40	C_6_H_6_O_3_	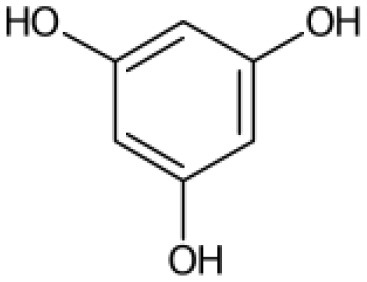	Benzofurans	Phloroglucinol
365.1050	153	C_12_H_22_O_11_	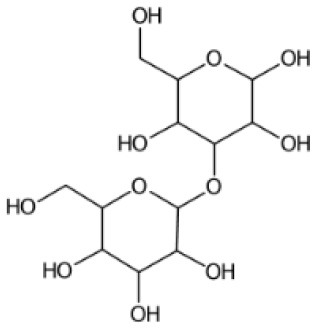	Organooxygen compounds	3-alpha-Mannobiose
310.3101	48	C_20_H_39_NO	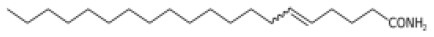	Fatty acyls	5-Eicosenamide

One larger cluster in the chemical similarity network in Figure 3 is the group of six glycerophospholipids, mainly phosphocholines and phosphatidylcholines, with two fatty acyl groups. Glycerophospholipids are integral to the membrane structure (Niu et al., 2013) and have also been found in the silica deposition vesicles that form the silica shells in the diatom *Thalassiosira pseudonana* (Schwarz et al., 2022). Enzymatic transformation of these metabolites, releasing choline, occurs through hydrolysis via phospholipase D, where choline acts as a downstream signaling molecule (Hausmann et al., 2011). Another reaction is the acyltransferase reaction, which catalyzes the release of fatty acids from the 1 or 2 acyl group position via phospholipase A (Duncan et al., 2008). These enzymatic activities have also been reported in diatoms (Pohnert, 2002; Beligni et al., 2015) but not with the glycerophospholipids found in this study. It is noteworthy that the spectral features annotated with these glycerophospholipids had smaller precursor masses as compared to the monoisotopic mass of the molecules, which can be attributed to the in-source fragmentation where one fatty acyl chain fragmented from the whole molecule, and so the annotations, in this case, are not extremely reliable.

Diatoms produce extracellular polysaccharides as part of their silica shell/frustules formed by monosaccharide building blocks. Generally, the cell wall of diatoms also contains mannose and glucuronic acid as main constituents and varying amounts of fucose and xylose (Gügi et al., 2015). A recent study showed that the cell wall of the diatom *Phaeodactylum tricornutum* contains α –(1 → 3)-mannan backbone, which could explain the presence of 3-alpha-mannobiose in diatoms as annotated with MAW (Le Costaouëc et al., 2017). Furthermore, xylose, maltotriose, and allulose were annotated with MAW and had a chemical similarity with three sugar molecules from the suspect list, arabinose, maltose, and sorbose, with a Tanimoto similarity score of 1. MAW annotated maltotriose, which is found in pennate diatom biofilms (Wigglesworth-Cooksey and Cooksey, 2005). Another monosaccharide allulose was annotated with MAW, which is rarely found in natural carbohydrate plants and macroalgae, with applications such as anti-atherosclerosis, anti-hyperlipidemic, and neuroprotective (Mu et al., 2018).

A few other metabolites annotated with MAW were phloroglucinol, malonamide, and two polyphenols (chlorogenate and 4-hydroxycinnamic acid). Phloroglucinol is a growth promoter produced by marine algae *Phaeophyceae* and *Fucaceae* and is known to increase the levels of the carotenoid pigment fucoxanthin in microalgae (Liu et al., n.d.). Malonamide is a dicarboxylic acid diamide, a derivative of malonic acid. Malonamide can be converted back to malonic acid via amidase. Malonamide derivatives (MAMDs) have been utilized as anticancer and antibiotic drugs (Purgatorio et al., 2022); however, not many studies have been conducted on the role of malonamide as a metabolite specifically in diatoms.

Diatoms, in general, are rich sources of polyphenolic compounds exhibiting antioxidant properties (Goiris et al., 2012). Studies show that diatom *Phaeodactylum tricornutum*, under copper stress, releases high concentrations of polyphenols such as 4-hydroxycinnamic acid and chlorogenic acid (Rico et al., 2013; Santiago-Díaz et al., 2023).

### 4.2 Prokaryote-associated metabolites

*S. marinoi* forms synergistic interactions with marine prokaryotes. The phycosphere of the marine diatom, which represents the diffusion-limited mucus-containing layer around the cells, hosts several bacterial species that stimulate the growth of the diatoms (Johansson et al., 2019). The bacteria provide the *S. marinoi* with vitamins and organic sulfur metabolites and get the nitrogen and carbon sources in return (Bruckner et al., 2011; Seymour et al., 2017). In this study, pure but not axenic *S. marinoi* strain was used for inoculation. *S. marinoi* grew with its associated microbiome that never became dominant in terms of biomass, as monitored by microscopy. Thus, we cannot rule out that some of the metabolites detected arise from metabolism by the associated bacteria, and hence, bacterial-produced metabolites were also measured and annotated. Here, we discuss a few high-scoring metabolites annotated with MAW reported in marine prokaryotes.

A sugar-based organic acid, gluconic acid, has been found in marine bacteria connected to the release of domoic acid as a toxin in blooms of the diatom *Pseudo-nitzschia multiseries* (Stewart et al., 1997). A benzenoid from the statin family, which is known for its therapeutic properties, fluostatin J, is generally found in marine actinobacteria (Zhang et al., 2012). Indole is produced by symbiotic bacteria supporting the growth of *Skeletonema marinoi* (Johansson et al., 2019). Marine bacteria and some marine algae execute the biodegradation of sulfoacetate to produce sulfoacetaldehyde, which is a major organic solute in marine organisms (Weinitschke et al., 2010). 6-Chloropurine riboside, derived from the bacterium *Geobacillus stearothermophilus*, is used as an antiviral and anti-tumoral therapeutic agent (Rivero et al., 2012).

### 4.3 Assessment of data quality and Metabolome Annotation Workflows

The metabolites annotated with MAW revealed a large number of chemical compounds from *Skeletonema marinoi*, which have also been previously reported in other diatoms. However, we also observed metabolites from prokaryotic, plant, and fungal origin (the latter two were discarded during the curation). Many of the metabolites from non-diatomic sources were also marine natural products, suggesting that both fungi and diatoms may produce similar compounds.

However, the annotation of certain spectral features to compounds of fungal origin could also be due to misannotation attributed to the limitations of mass spectrometry techniques and current annotation tools and databases and the complexity of chemical data. Mass spectrometry (MS) is currently the main viable option for untargeted metabolomics, followed by manual curation and verification (Chaleckis et al., 2019). The MS-based search for metabolite annotation rarely leads to unique biochemical structures due to the presence of compound isomers in the databases used for dereplication and the limitations in the mass accuracy of the MS system used (Xiao et al., 2012). The MS^1^ peaks chosen for MS^2^ fragmentation have varying optimal fragmentation energy, so not all MS^2^ spectra are informative. Features obtained with an untargeted LC-MS^2^ approach, which do not have an MSI level 1 confidence score (based on the known internal standard measurement), cannot provide absolute certainty about the assigned chemical structure for a feature due to the lack of our current knowledge of the chemical dark space. Moreover, the current databases also only favor well-studied model organisms and may not cover the entire metabolome of *S. marinoi*, which is why it is important to elucidate the metabolome of this diatom.

To overcome a fraction of these challenges, MAW automatized the annotation process and performed dereplication in both spectral and compound databases while integrating prior biochemical knowledge in the form of a suspect list, which is also important for making accurate annotations. To give each annotation a confidence score, the Metabolomics Standards Initiative (MSI) levels of confidence were implemented in MAW to follow the standards set by the metabolomics scientific community. Automated workflows, such as MAW, aid in the standardization and reproducibility and reduce the manual work in the analysis. Metabolomics is a relatively new omics field, and the standardization in metabolomics is not widely adopted, which leads to missing metadata and reproducibility issues. However, it is challenging to follow a set of standards in metabolomics because of the different combinations of chromatography and data acquisition methods of a diverse set of metabolites. Moreover, although using ChemONT, which is a standardized vocabulary for chemical classification, one metabolite with a carboxylic group and an amine group can be categorized as either an organic acid or an organic nitrogen compound. As of now, MAW follows the currently available standards and ontologies for the spectral data acquired from data-dependent acquisition (DDA) LC-MS^2^ data, associating one chemical class to each annotation.

### 4.4 Future perspective

The catalog of metabolites presented in this study exhibits a wide range of coverage provided by untargeted LC-MS-metabolomics. However, this approach does not provide validity for structure identification. To overcome this limitation, it is recommended to conduct targeted studies using some of the biologically important NPs. To further improve the accuracy of metabolite annotation in future studies, techniques such as nuclear magnetic resonance (NMR) spectroscopy can be employed to provide additional structural information for metabolite identification. The development of more comprehensive annotation tools and submission of new experimental spectra to natural product databases like GNPS will facilitate better metabolite annotation and identification, contributing to the expansion of available metabolites. Re-annotation with new updates of the databases can also enhance the annotation results. An integrative study of genomics and metabolomics from this diatom can also reveal the production of secondary metabolites involved in different biomechanisms in the marine ecosystem, providing a systems-level understanding. Being the model organism for diatoms, this could hold the potential for elucidating biochemical mechanisms in other diatom species and unveiling marine-centric metabolic pathways (Edison et al., 2016).

## Data availability statement

The datasets presented in this study can be found in online repositories. The RAW and mzML metabolome data and the identifications can be found here: European Molecular Biology Laboratory's European Bioinformatics Institute (EMBL-EBI) MetaboLights, https://www.ebi.ac.uk/metabolights/, MTBLS2892 (Haug et al., 2020). The mzML files can be found here: Zenodo, https://zenodo.org/, 10.5281/zenodo.7515842 and 10.5281/zenodo.7515829. The currently available versions of GNPS, HMDB, and MassBank can be found here: Zenodo, https://zenodo.org/, 10.5281/zenodo.6528931. The suspect list for *Skeletonema* spp. can be found here: Zenodo, https://zenodo.org/, 10.5281/zenodo.5772755. The scripts used to generate the list of metabolites using MAW version 1.1 and for the suspect list curation can be found here: GitHub, https://github.com/, zmahnoor14/MAW-Diatom. The list of annotated metabolites from MAW, intersection and union lists, along with the corresponding sunburst plots can be found here: Zenodo, https://zenodo.org/, 10.5281/zenodo.7798782.

## Author contributions

MZ: Data curation, Formal analysis, Methodology, Software, Validation, Writing—original draft, Writing—review & editing. DS: Formal analysis, Investigation, Methodology, Writing—original draft, Writing—review & editing. SS: Formal analysis, Methodology, Writing—review & editing. VN: Formal analysis, Methodology, Writing—review & editing. GP: Conceptualization, Funding acquisition, Project administration, Resources, Supervision, Writing—review & editing. CS: Conceptualization, Funding acquisition, Project administration, Resources, Supervision, Writing—review & editing. KP: Project administration, Supervision, Writing—review & editing. MS: Conceptualization, Investigation, Project administration, Supervision, Writing—review & editing.
