# Supplementary material for: Untargeted metabolomics to expand the chemical space of the marine diatom Skeletonema marinoi
Source: Front Microbiol. 2023 Dec 5;14:1295994. doi: 10.3389/fmicb.2023.1295994 (PMC10728474; doi:10.3389/fmicb.2023.1295994)
Supplement: Supplementary file 1 [file Data_Sheet_1.PDF]

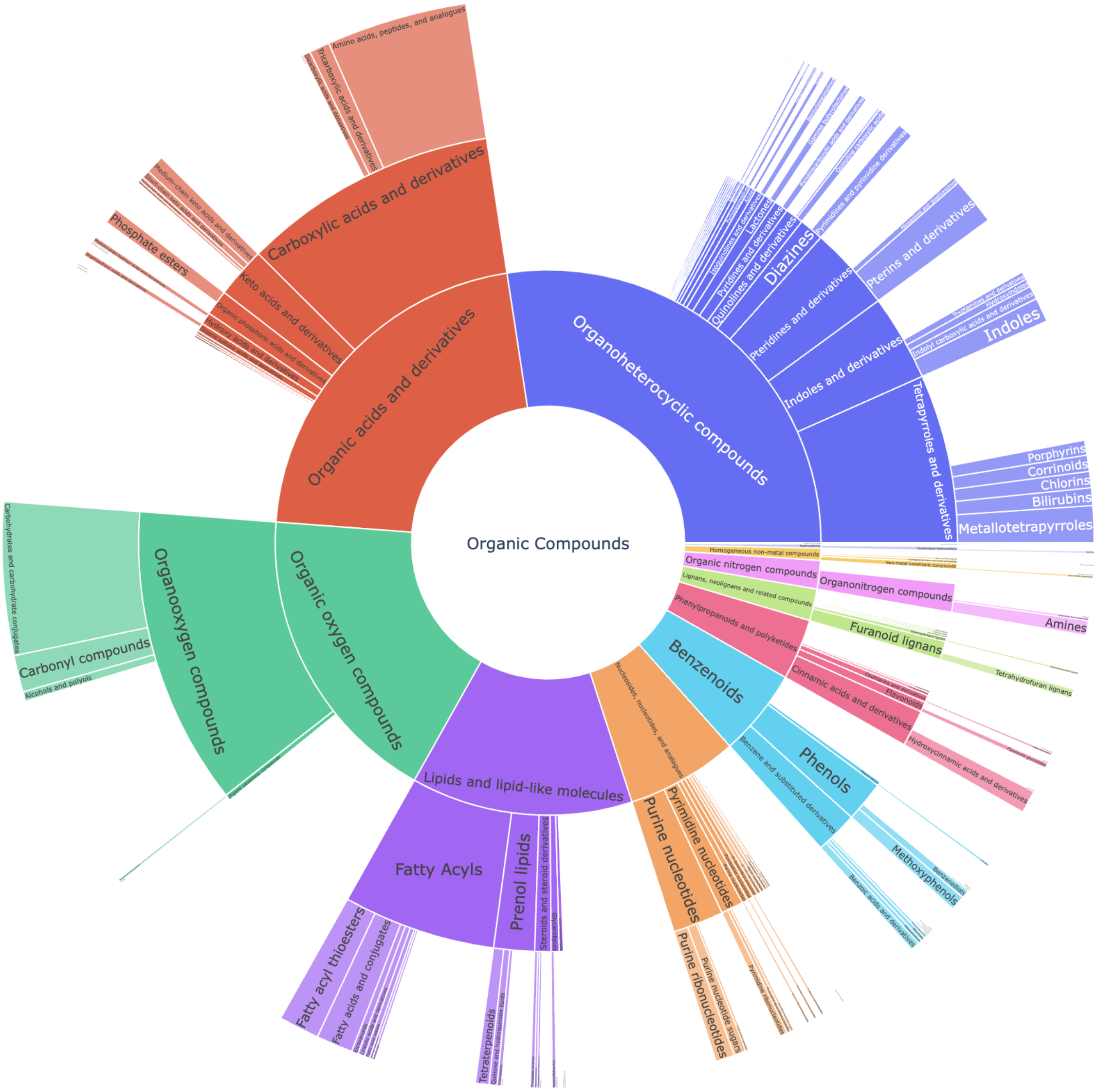

Figure S1: Sunburst plot for chemical classification of the known metabolites (compounds in the suspect list) using ClassyFire. The innermost circle represents the chemical compound kingdom of organic compounds, which is then classified into superclasses, classes, and subclasses. Among the compounds in the suspect list, the most prevalent superclasses are Organoheterocyclic compounds (27.7%), Organic acids and derivatives (21%), Organic oxygen compounds (17.5%), and Lipids and lipid-like molecules (12.8%).

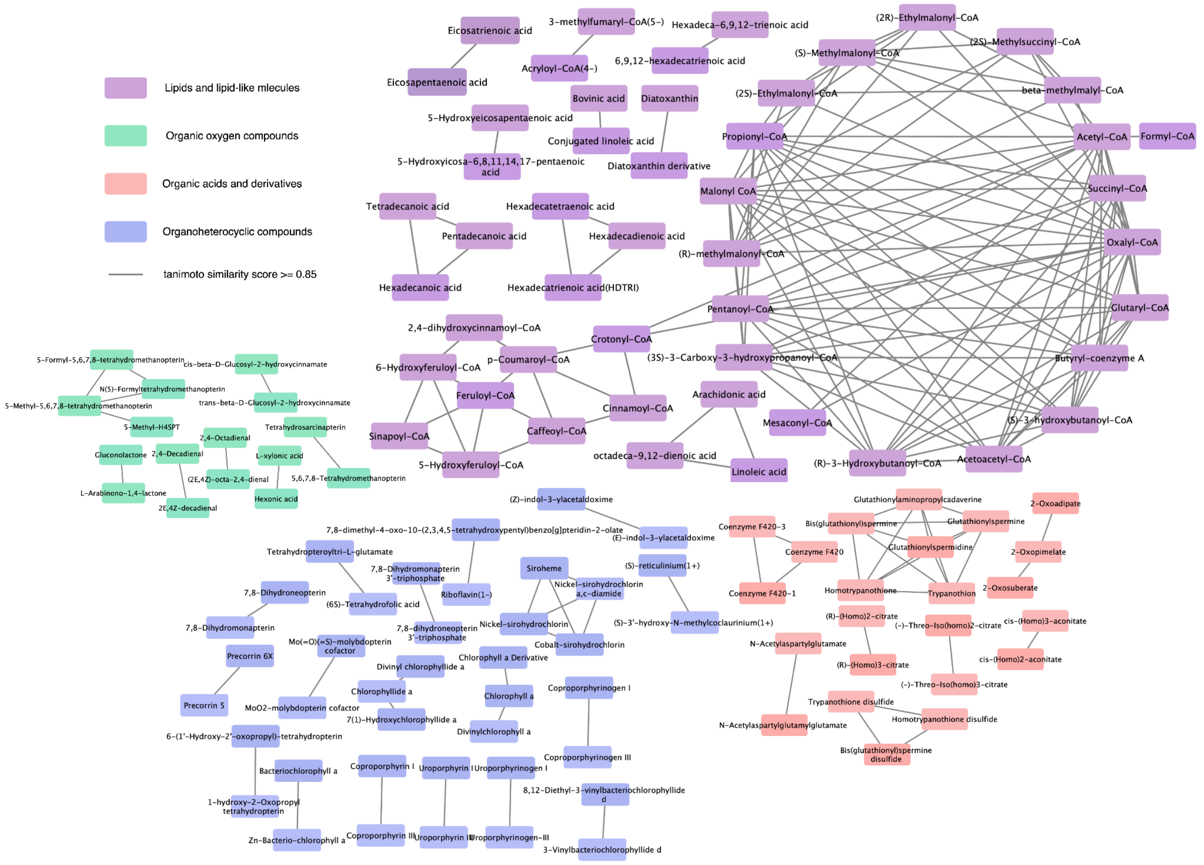

Figure S2: Chemical structure similarity network from compounds present in the suspect list. This network represents the chemical similarity among the most abundant superclasses Organoheterocyclic compounds, Organic acids and derivatives, Organic oxygen compounds, and Lipids and lipid-like molecules (being the most chemically similar group). The chemical similarity is determined with the Tanimoto similarity score >= 0.85.

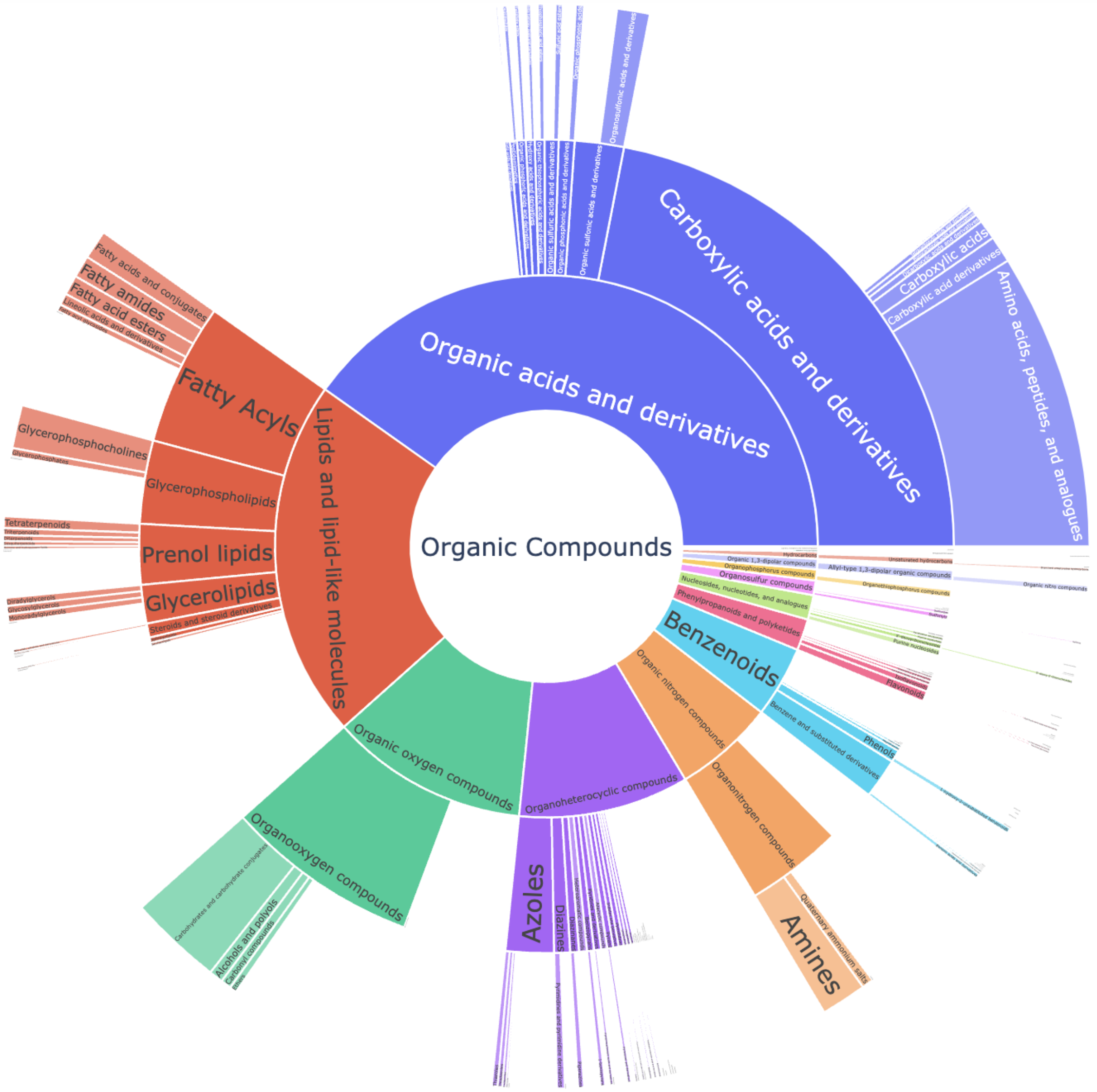

Figure S3: Sunburst plot for chemical classification of compound annotations from *S. marinoi* data. The plot shows the kingdom (Organic compounds), superclasses, classes, and subclasses of the compounds. In the LCMS2 data from *S. marinoi*, most of the compounds belonged to Organic acids and derivatives (37.5%), Lipids and lipid-like molecules (21.2%), Organic oxygen compounds (11.2%), and Organoheterocyclic compounds (11.9%).

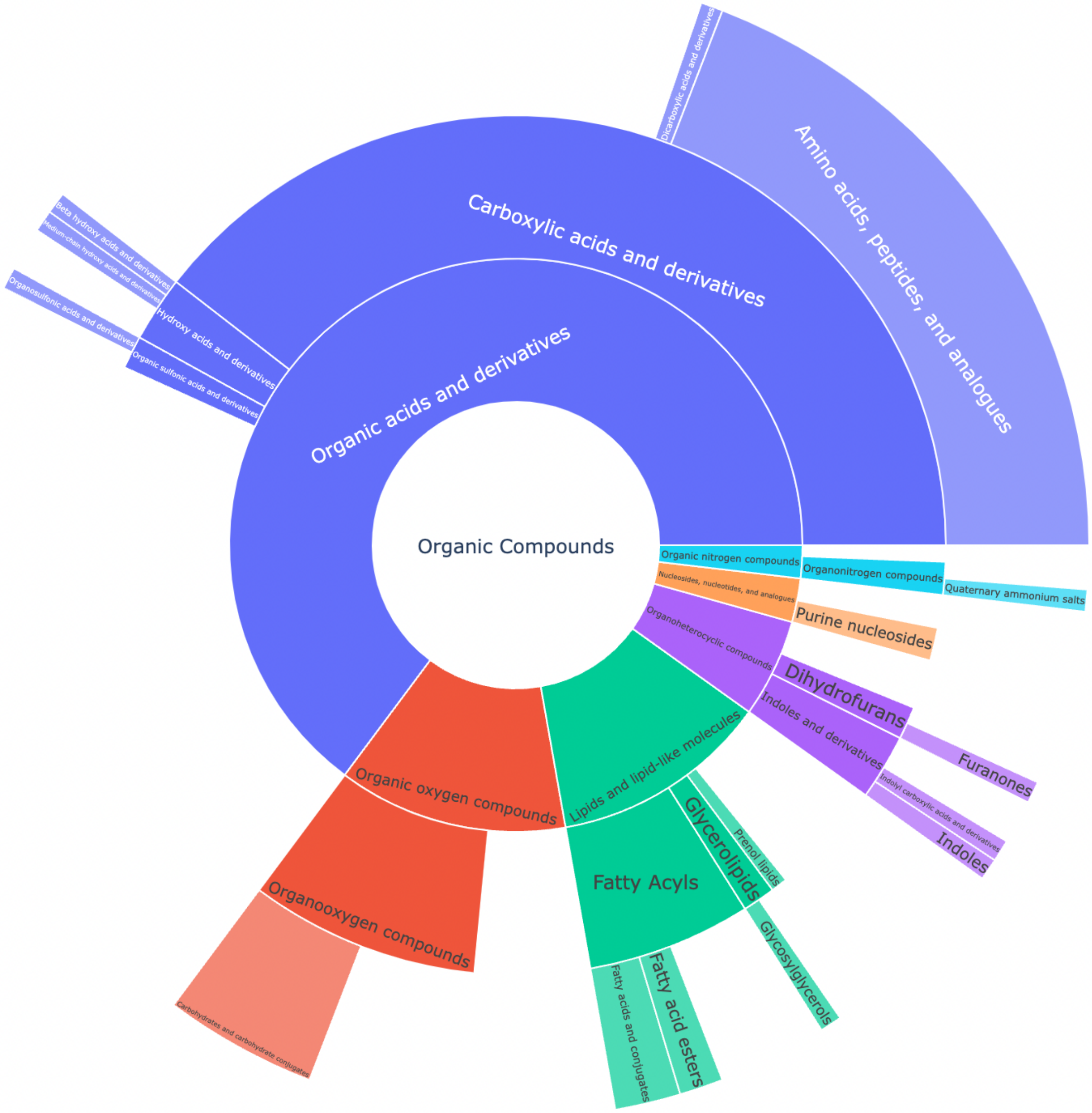

Figure S4: Sunburst plot for chemical classification of the common metabolites between the suspect list and the LC-MS² data of *S. marinoi* by MAW. Among the compounds in the suspect list, the most prevalent superclasses are, Organic acids and derivatives (63.6%), Organic oxygen compounds (12.7%), Lipids and lipid-like molecules (12.7%), and Organoheterocyclic compounds (5.4%)
